# Effects of a Dual Task of Exercise and Inhibitory Training on Cognitive Control Toward Food Cues and Frontal EEG Activity

**DOI:** 10.3390/brainsci16080855

**Published:** 2026-08-13

**Authors:** Jeong-In Gong, Seo-Hyun Yoo, Jin-Hyuk Eom, Sung-Yeon Oh, Dong-Yeop Lee, Ji-Heon Hong, Jae-Ho Yu, Jeong-Woo Jeon, Yeon-Gyo Nam

**Affiliations:** Department of Physical Therapy, College of Health Sciences, Sunmoon University, Asan-si 31460, Republic of Korea; gji0821@naver.com (J.-I.G.); seohyun327@naver.com (S.-H.Y.); jimmyeom0114@gmail.com (J.-H.E.); akk1263@naver.com (S.-Y.O.); leedy@sunmoon.ac.kr (D.-Y.L.); hgh1020@hanmail.net (J.-H.H.); naresa@sunmoon.ac.kr (J.-H.Y.);

**Keywords:** dual-task intervention, moderate-intensity exercise, EEG, inhibitory control, subjective appetite

## Abstract

Background/Objectives: This study aimed to examine the acute effects of a dual-task intervention combining moderate-intensity cycling with a food-related Go/No-Go task on inhibitory control, subjective appetite, and frontal electroencephalogram (EEG) activity in healthy adults. Methods: Forty healthy adults were randomly assigned to an experimental group or a control group in a single-blind design. The experimental group performed cycling combined with a food-related Go/No-Go task, whereas the control group performed only the cognitive task. Outcomes were assessed before and after the intervention, and Group × Time effects were analyzed using repeated-measures ANOVA with correction for multiple comparisons. Results: No significant Group × Time interactions were observed for the primary outcomes, including EEG alpha and theta power (ηp^2^ = 0.004 and 0.040, respectively) and Stroop reaction time and accuracy (ηp^2^ = 0.076 and 0.062, respectively). Subjective appetite decreased in the experimental group and increased in the control group, showing a significant interaction at the uncorrected level (*p* = 0.017, ηp^2^ = 0.142); however, this interaction did not remain significant after correction for multiple comparisons. The exploratory DEBQ outcomes showed no significant Group × Time interactions. Conclusions: In this single-session study, the intervention did not demonstrate clear intervention-specific benefits across the cognitive, EEG, or eating-related outcomes. Nevertheless, the contrasting descriptive pattern in subjective appetite warrants further investigation in larger studies involving repeated interventions and clinical populations.

## 1. Introduction

Obesity is medically defined as a condition characterized by excessive body fat accumulation that negatively affects an individual’s health [1]. It is commonly assessed by Body Mass Index (BMI), where individuals with a BMI of 30 kg/m^2^ or higher are classified as obese [1]. Globally, over 1.9 billion adults aged 18 years and older are overweight, and more than 650 million are obese [1]. Obesity significantly increases the risk for numerous chronic health conditions, including type 2 diabetes mellitus, cardiovascular diseases, hypertension, stroke, certain types of cancers such as colorectal and breast cancer, and musculoskeletal disorders like osteoarthritis [2,3]. Obesity results from complex interactions among genetic susceptibility, metabolic regulation, and environmental influences [4]. While genetic predisposition plays an important role, the dramatic rise in global obesity rates over the past few decades suggests that environmental and behavioral factors are the predominant drivers [4,5].

In modern society, the proliferation of food delivery services, 24 h convenience stores, and social media-driven food marketing has markedly increased both the accessibility and omnipresence of high-calorie foods. Consequently, individuals are frequently exposed to visual and sensory food cues outside of traditional mealtimes, which contributes to more frequent impulsive eating and unplanned food consumption [6,7]. These behaviors have been consistently linked to diminished cognitive control and weakened inhibitory processes—both of which play a crucial role in regulating goal-directed behavior and resisting temptations [4]. A lack of inhibitory control increases susceptibility to indulging in high-calorie foods [8]. Several experimental studies have demonstrated that exposure to high-calorie food images can impair performance on cognitive control tasks, such as the Stroop Test, and alter frontal theta activity associated with conflict control [9].

This deficit in inhibitory control represents a significant pathway to obesity, as individuals struggle to resist impulses toward high-calorie food consumption despite being aware of the negative health consequences [4,5]. To address this deficit, acute aerobic exercise at moderate to high intensity has been reported to temporarily improve inhibitory control, attention, and working memory [5,10], whereas Response inhibition training using tasks such as the Go/No-Go paradigm may improve inhibitory control [8,11]. However, the evidence has not been uniformly positive. A systematic review and meta-analysis found no significant pooled long-term effect of response inhibition training on cognitive function in healthy adults, while stop-signal training alone also showed no significant immediate effect [11]. Studies combining physical exercise with cognitive training have also reported potential benefits for executive function and neural activity [12,13,14]. Nevertheless, one acute study found that simultaneous physical and cognitive exercise was not superior to physical or cognitive exercise alone in improving executive function or prefrontal cortex oxygenation [12].

In addition, previous combined interventions have generally used non-food-specific cognitive tasks and have often focused on older adults rather than food-related inhibitory control in healthy young adults [12,13,14]. To our knowledge, few studies have directly compared food-related inhibitory training performed with and without simultaneous exercise, leaving it unclear whether adding moderate-intensity exercise produces acute effects beyond those of food-related inhibitory training alone [5,11,12,13,14]. Furthermore, studies simultaneously examining behavioral performance, subjective appetite, eating-related responses, and EEG activity within the same food-cue paradigm remain scarce [5,9,11,12,13,14].

Accordingly, the present study examined whether adding moderate-intensity cycling to a food-related Go/No-Go task produced greater acute changes in inhibitory control, frontal EEG activity, subjective appetite, and eating-related responses than the food-related Go/No-Go task alone in healthy young adults. By incorporating food-specific visual stimuli (e.g., high-calorie snacks, desserts, and fast food) into the dual-task design, this study aims to enhance ecological validity and better reflect everyday dietary temptations. General inhibitory control will be assessed using the Stroop Test [15], while eating behavior and appetite will be measured with the Dutch Eating Behavior Questionnaire (DEBQ) [16] and Visual Analogue Scale (VAS) [17].

The primary outcomes were Stroop performance, assessed using reaction time and accuracy, and frontal EEG activity, assessed using alpha and theta power. The secondary outcomes were subjective appetite, assessed using the Visual Analogue Scale (VAS), and self-reported eating behavior, assessed using the Dutch Eating Behavior Questionnaire (DEBQ). We hypothesized that, compared with the control group, the experimental group would show greater improvements in the primary outcomes, including shorter Stroop reaction times, higher Stroop accuracy, increased mean EEG alpha power, and decreased mean EEG theta power. Among the secondary outcomes, we hypothesized that the experimental group would show a greater reduction in subjective appetite. Changes in restrained, emotional, and external eating scores measured using the DEBQ were examined as exploratory secondary outcomes without a prespecified directional hypothesis.

## 2. Materials and Methods

### 2.1. Study Design

This study employed a randomized, single-blind, controlled pretest–posttest design. The study protocol was approved by the Institutional Review Board of Dongbang Culture Graduate University (IRB No. 8223-202601-HR-001-01). All participants were informed of the purpose and procedures of the study and provided voluntary informed consent before participation. This trial was registered at the Clinical Research Information Service (CRIS), Republic of Korea (registration no. KCT0012295; retrospectively registered). Prospective registration was not completed before study initiation because the need for trial registration was identified only during the journal’s editorial review process.

### 2.2. Participants

This study was conducted among healthy adults of both sexes. The general characteristics of the participants are presented in Table 1. Forty eligible participants were randomly allocated to the experimental or control group in a 1:1 ratio (*n* = 20 per group). The exclusion criteria were as follows: individuals taking medications that could affect EEG results, those with cardiovascular or respiratory diseases that make moderate-to-high intensity exercise inappropriate, and individuals with visual color vision deficiency [18].

### 2.3. Experimental Procedures

The study was conducted as a single-session, single-blind randomized controlled experiment. After all 40 eligible participants had been recruited and enrolled, a researcher who had not participated in participant recruitment generated the randomization sequence using the RAND and RANK functions in Microsoft Excel and implemented the group assignments. Because the sequence was generated only after enrollment was completed, allocation information was unavailable during recruitment, and no separate allocation-concealment procedure was used. The researchers conducting the experimental procedures were aware of the participants’ group assignments. Written informed consent was obtained from all participants before participation. Although participants were aware of the activities they personally performed, they were not informed of the alternative intervention condition, whether their assigned condition was designated as the experimental or control condition, or the directional study hypothesis. Thus, in the present study, single blinding referred specifically to participant blinding regarding the alternative intervention condition, group designation, and directional study hypothesis. Participants were scheduled individually and had no contact with other participants during the experimental procedures. Participants were instructed to consume a meal 1–2 h before testing and to avoid arriving in a fasted state.

**Figure 1:**
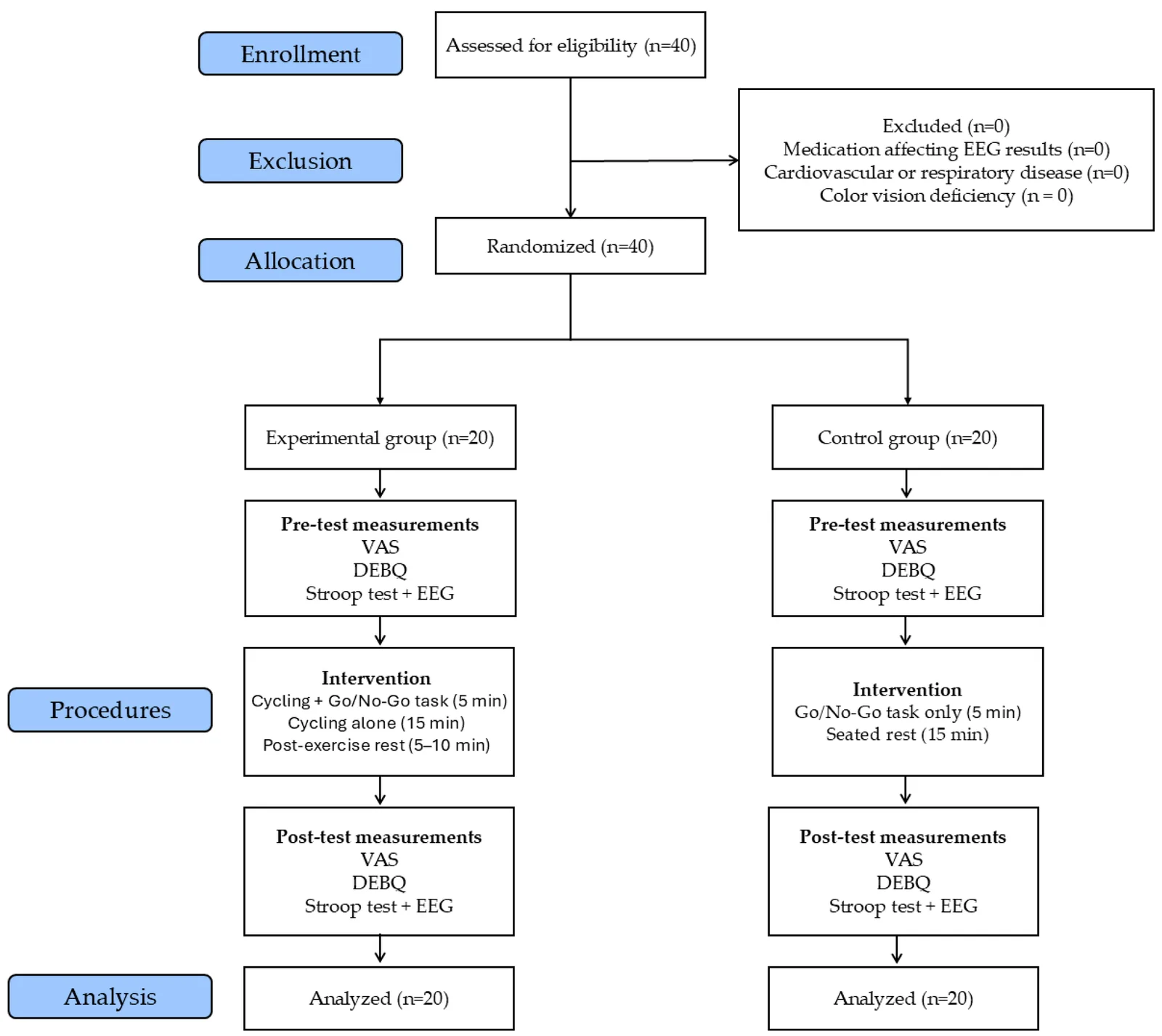
Study flowchart showing the sequence and duration of each intervention phase.

Before the experiment, the participants’ weight, height and age were accurately recorded. Subsequently, eating behavior-related responses were collected using the Dutch Eating Behavior Questionnaire (DEBQ) [16] and current appetite status was measured using the Visual Analogue Scale (VAS) [17,19]. While performing the Stroop Test, electroencephalogram (EEG) signals were recorded, and band-power analysis was used to quantify alpha and theta activity [20]. To ensure accurate EEG measurements, participants were instructed to maintain a clean scalp prior to the experiment, avoiding the use of cosmetics, hair wax, and other similar substances. Prior to electrode placement, the scalp areas were thoroughly cleaned to remove any foreign materials. This procedure was necessary because contaminants on the scalp can increase impedance, thereby interfering with the accurate detection of electrical signals generated by the brain. Additionally, the surrounding environment was kept quiet to minimize artifacts and prevent EEG signal disruption. The intervention consisted of a simultaneous cycling exercise and Go/No-Go task [21]. After the intervention, DEBQ and VAS were re-administered and EEG (alpha and theta waves) was again recorded during the Stroop Test (Figure 1).

### 2.4. Intervention

#### 2.4.1. Experimental Group: Go/No-Go Task + Cycling

**Figure 2:**
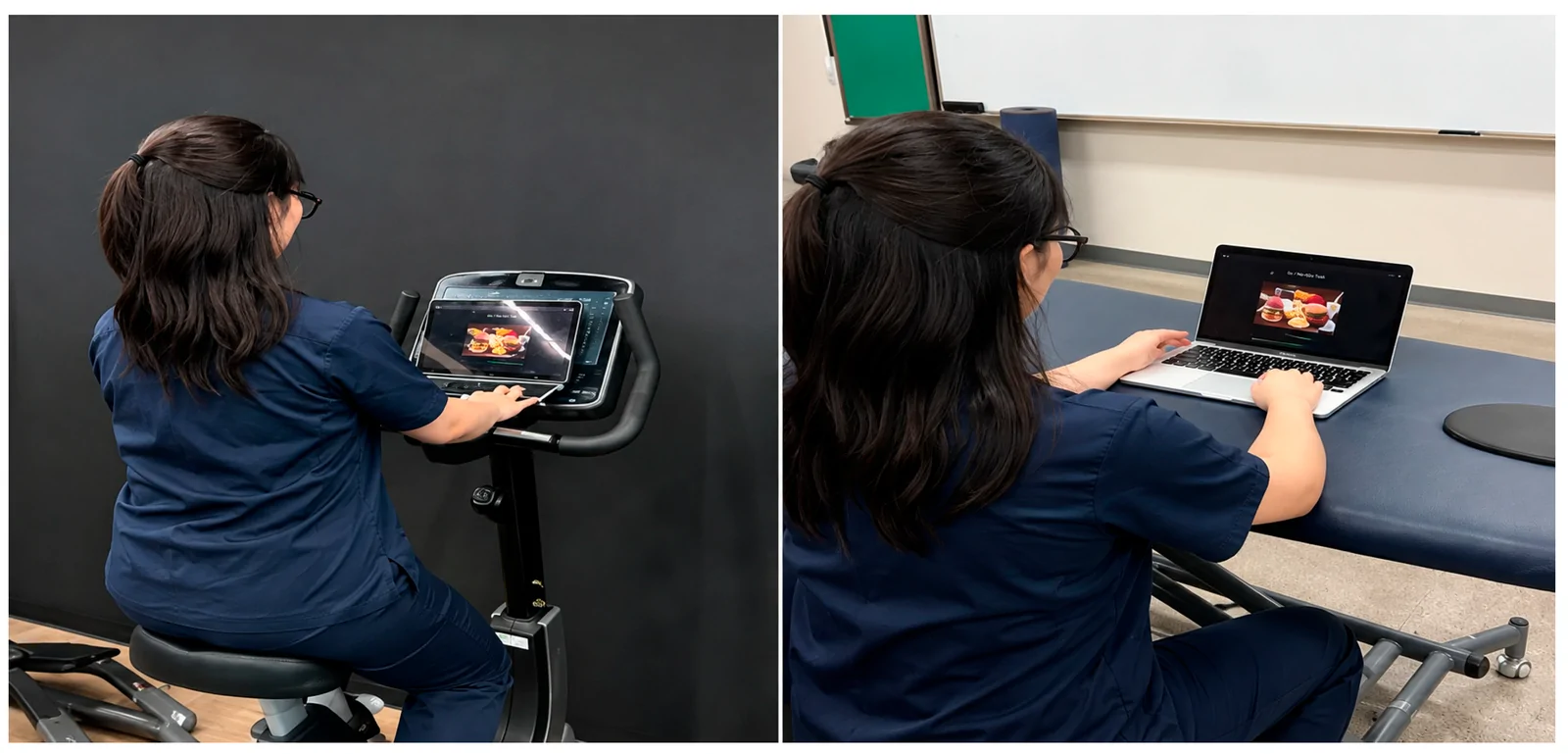
Go/No-Go Task + Cycling (**Left**), Go/No-Go Task Only (**Right**).

The experimental group underwent a dual-task intervention after the pre-test measurements that combined cycling exercise with a Go/No-Go task. Participants began cycling and the food-related Go/No-Go task simultaneously, without a separate cycling period before the task to stabilize exercise intensity. The target exercise intensity was set at a perceived exertion level of approximately 12 (RPE 12) [21]. During the 5 min dual-task period, the researcher repeatedly assessed participants’ perceived exertion and adjusted the cycle resistance to reach and maintain the target intensity. If the target RPE could not be achieved by increasing resistance alone, participants were instructed to increase their pedaling cadence. Heart rate, actual cycle resistance, and workload were not objectively recorded. Participants simultaneously performed the Go/No-Go task for 5 min while riding a stationary bicycle (fixed-type model). The task was displayed on a 15-inch laptop screen, and participants responded by pressing the space bar key using their preferred hand. After completing the cognitive task, they continued cycling alone for an additional 15 min without any cognitive load. To ensure consistency in measurement, participants were not exposed to any additional visual or auditory food-related stimuli beyond those presented as part of the Go/No-Go task. After the exercise session, the experimental group was given an additional 5–10 min of rest, during which participants were allowed to cool down and remove sweat or foreign substances from their scalp. This procedure aimed to minimize potential changes in EEG values caused by perspiration (Figure 2).

#### 2.4.2. Control Group: Go/No-Go Task Only

The control group performed the Go/No-Go task alone for 5 min without any physical activity. The task was presented on a 15-inch laptop screen, and participants responded with the space bar key using their preferred hand. After completing the task, participants remained seated and rested for 15 min. To ensure consistency in measurement, participants were not exposed to any additional visual or auditory food-related stimuli beyond those presented as part of the Go/No-Go task (Figure 2).

### 2.5. Measures

#### 2.5.1. Stroop Test

**Figure 3:**
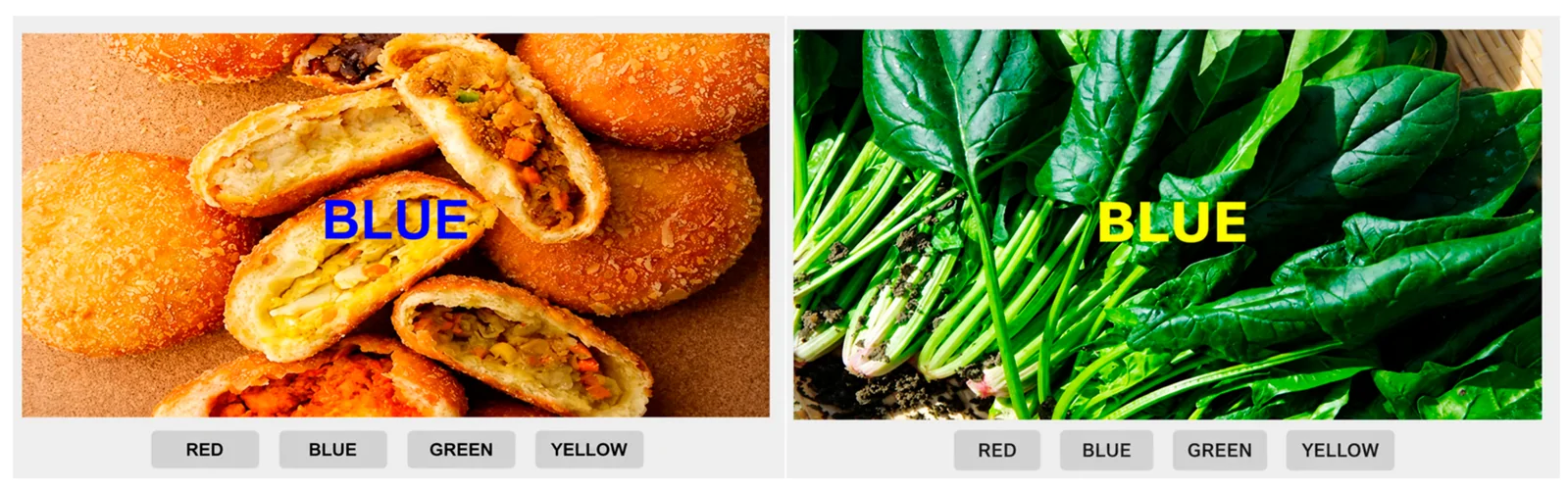
Stroop Test.

In this study, the Go/No-Go task was used as the intervention task, whereas the Stroop Test was used as an outcome measure to assess inhibitory control before and after the intervention. The Stroop Test was conducted while participants were wearing the EEG device. On each trial, an image of either a high-calorie or low-calorie food was randomly presented as the background. A color word appeared in the center of the screen, and the font color of the word was randomly presented in an incongruent manner with the meaning of the word itself (e.g., red, blue, green, or yellow). Participants were instructed to identify the color of the text, not the meaning of the word. Participants responded using a computer mouse. Upon completion of the test, the final screen displayed the accuracy rate and average response time for each calorie condition (Figure 3).

#### 2.5.2. Dutch Eating Behavior Questionnaire (DEBQ)

After completing the VAS, the DEBQ was administered as an exploratory secondary measure to assess restrained, emotional, and external eating tendencies. Participants completed the DEBQ while EEG electrodes were being attached prior to the Stroop Test, which required approximately 2–3 min. The DEBQ consists of 33 items and measures three distinct eating behaviors [16]: These include restrained eating (items 1–10), which assesses behaviors associated with weight control, dieting, and restriction of food intake; emotional eating (items 11–23), which evaluates the tendency to overeat or snack in response to emotional states such as anxiety, depression, stress, loneliness, or anger; and external eating (items 24–33), which measures the tendency to initiate eating in response to external food cues rather than internal hunger signals. Each item was rated on a five-point Likert scale, ranging from “Never” (1) to “Very often” (5). Total scores could range from 33 to 165, with higher scores indicating stronger tendencies toward the respective eating behaviors. The DEBQ has been translated into Korean.

#### 2.5.3. Visual Analogue Scale

**Figure 4:**
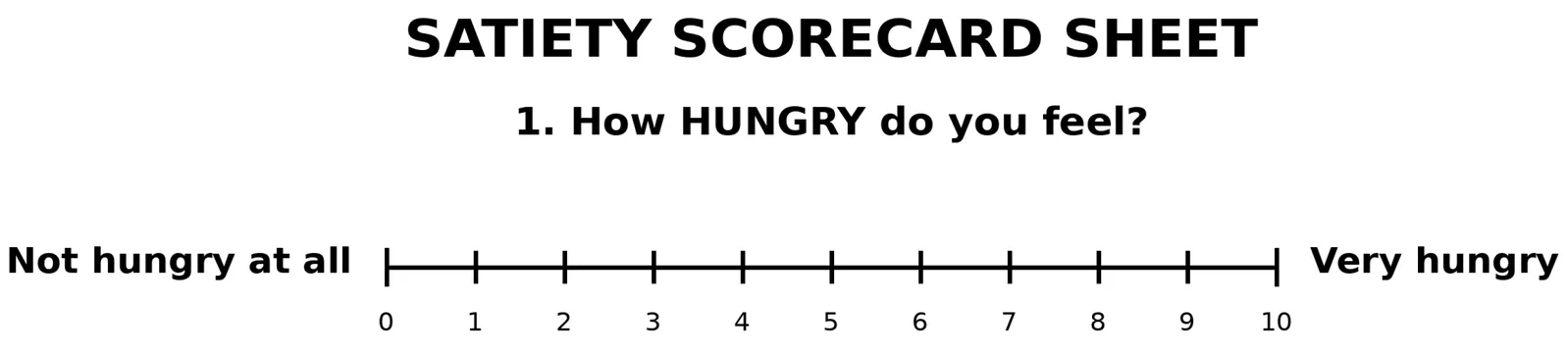
Visual Analog Scale.

VAS was first measured as soon as participants entered the room, before the DEBQ was administered. After completion of the intervention and the subsequent rest period, the VAS was administered first, followed by the DEBQ, while EEG electrodes were being attached in both groups. The VAS was used to assess subjective appetite perception, specifically hunger [17,19]. Previous acute-exercise studies have also used subjective appetite measures to evaluate short-term appetite responses [22]. Participants rated their hunger level on a 0–10 scale, where 0 indicated “not hungry at all” and 10 indicated “extremely hungry.” Higher scores indicated greater subjective hunger. The same 0–10 VAS assessment procedure was used for all participants [17] (Figure 4).

#### 2.5.4. Alpha and Theta Waves

**Figure 5:**
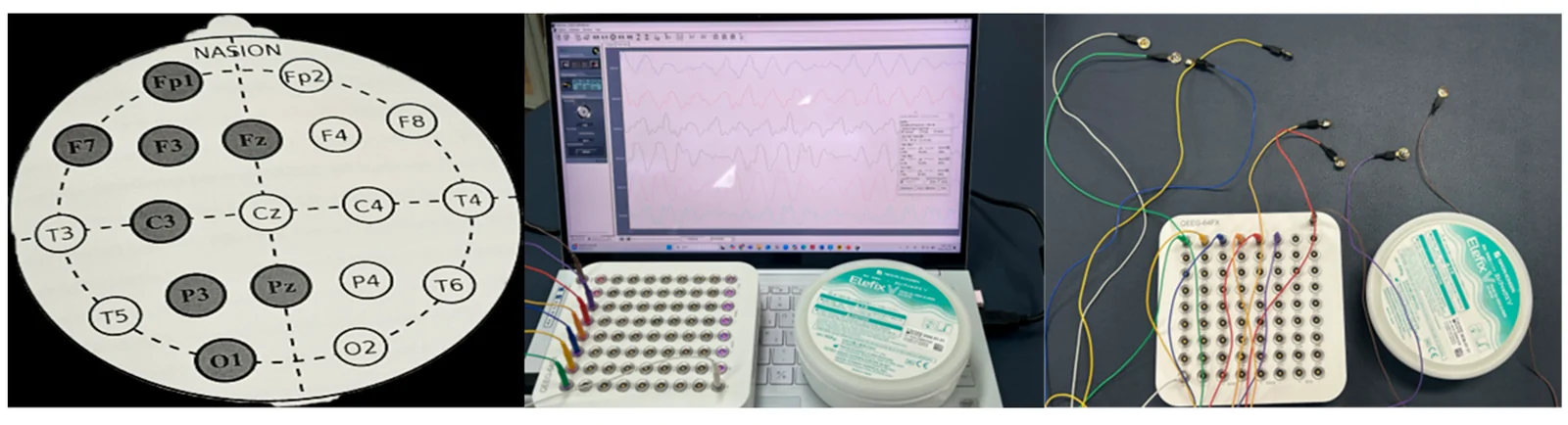
EEG recording device and electrode channel configuration.

EEG was recorded simultaneously during the Stroop Test to examine changes in brain activity related to inhibitory control and executive functioning [23]. Previous studies have used EEG-derived measures to examine neural responses during food-related inhibitory control tasks [9,24]. The Stroop Test contained pictures of 30 high-calorie foods and 30 low-calorie foods, and participants were required to select the color of the word that was written at the center of the picture. EEG data were recorded using an 8-channel EEG device (Laxtha Inc., Daejeon, Republic of Korea) (Figure 5). Six scalp channels were placed over the frontal (F3, F4, and Fz), parietal (Pz), and occipital (O1 and O2) regions, and A1 and A2 were attached to the bilateral mastoid processes as reference electrodes. EEG signals were recorded at 250 Hz and analyzed using TeleScan software (CD-TS-2.2; LAXTHA Inc., Daejeon, Republic of Korea). During EEG recording, participants were instructed to maintain an upright posture and minimize unnecessary body and facial movements. After applying a 0.5–50 Hz band-pass filter and a 60 Hz notch filter, each recording was visually inspected. Because unstable waveforms were frequently observed at the beginning and end of the recordings, these portions were excluded. To standardize the analyzed duration across participants and measurement time points, approximately 40 s of relatively stable EEG data without visually apparent excessive noise were manually selected from each recording using the same visual criteria. Power spectral analysis was performed, and relative power values for alpha (8–13 Hz) and theta (4–7 Hz) extracted from the six channels were averaged and natural-log transformed before statistical analysis. The preprocessing protocol relied on visual inspection and manual selection of a standardized 40 s stable segment rather than automated artifact correction. This approach was considered adequate for the present exploratory analysis because the same segment duration and visual criteria were applied across all participants and measurement time points, although residual artifacts could not be completely excluded. The transformed mean alpha and theta power values were used for the pre- and post-intervention comparisons.

This dual-task methodology was chosen based on previous studies suggesting that combined physical and cognitive tasks may influence cognitive functioning and prefrontal activity [12,13,14]. Related studies have also examined factors affecting neural responses to food cues and cognitive–motor interference during dual-task performance [25,26].

### 2.6. Statistical Analysis

All statistical analyses in this study were performed using the SPSS 29.0 statistical software program. Mean and standard deviation were calculated for each measurement. The required sample size was calculated a priori using G*Power 3.1 for the originally planned analysis. Because no directly comparable previous study was available, a large anticipated standardized effect size of 0.80 was assumed. With an alpha level of 0.05 and a statistical power of 0.80, the minimum required sample size was estimated to be 34 participants. Considering possible dropouts, a total of 40 participants were recruited. The value of 0.80 was not derived from previous literature. At the time of study planning, no published study had examined the acute effects of a dual task combining moderate-intensity cycling with a food-related Go/No-Go task on inhibitory control or frontal EEG activity, and no comparable effect-size estimate was therefore available; a conventional large effect was assumed in the absence of such data. We acknowledge that this assumption was optimistic. To provide a more informative basis for interpreting the present results, a post hoc sensitivity analysis was conducted using the realized sample of 40 participants (20 per group). With an alpha level of 0.05, a statistical power of 0.80, two groups, two measurement points, and an assumed correlation of 0.50 among repeated measures, the smallest Group × Time interaction that the study could reliably detect was f = 0.23 (ηp^2^ ≈ 0.05); interaction effects smaller than this magnitude could not be detected with adequate power. Normality was assessed prior to the inferential analyses. EEG power values were natural-log transformed prior to analysis to reduce distributional skewness [27]. Group × Time interactions were tested using two-way repeated-measures ANOVA (within-subjects factor: Time [pre, post]; between-subjects factor: Group [experimental, control]). Stroop reaction time and accuracy were analysed with a three-way (Group × Time × Calorie) repeated-measures ANOVA. Effect sizes are reported as partial eta-squared (ηp^2^). To control the family-wise Type I error rate across the eight Group × Time interaction tests, a Bonferroni correction was applied (α = 0.05/8 = 0.00625).

## 3. Results

EEG analysis revealed significant main effects of Time for both alpha and theta power. For alpha power, the Group × Time interaction was not significant (F = 0.159, *p* = 0.693, ηp^2^ = 0.004). A significant main effect of Time was observed (F = 8.767, *p* = 0.005), whereas the main effect of Group was not significant (F = 3.753, *p* = 0.060). Alpha power increased from pre- to post-test in both the control group (−1.697 ± 0.224 to −1.610 ± 0.164) and the experimental group (−1.619 ± 0.205 to −1.505 ± 0.130). For theta power, the Group × Time interaction was also not significant (F = 1.592, *p* = 0.215, ηp^2^ = 0.040). A significant main effect of Time was observed (F = 14.946, *p* < 0.001), whereas the main effect of Group was not significant (F = 0.819, *p* = 0.371). Theta power decreased from pre- to post-test in both the control group (−1.291 ± 0.342 to −1.426 ± 0.246) and the experimental group (−1.305 ± 0.392 to −1.571 ± 0.283). Thus, although alpha and theta power changed over time, the magnitude of these changes did not differ significantly between the groups (Table 2).

Stroop Test analysis revealed a significant main effect of Time for reaction time (F = 67.687, *p* < 0.001). However, the Group × Time interaction was not significant (F = 3.139, *p* = 0.084, ηp^2^ = 0.076), and the main effect of Group was also not significant (F = 0.001, *p* = 0.970). Reaction time decreased from pre- to post-test in both groups under both the low-calorie and high-calorie conditions, but the magnitude of the reduction did not differ significantly between the groups. For Stroop accuracy, the Group × Time interaction was not significant (F = 2.496, *p* = 0.122, ηp^2^ = 0.062), and the main effect of Time was also not significant (F = 2.496, *p* = 0.122). A significant main effect of Group was observed (F = 5.621, *p* = 0.023), indicating an overall difference in accuracy between the groups across time points and calorie conditions; however, changes in accuracy over time did not differ significantly between the groups. Neither the main effect of Calorie nor any interaction involving Calorie was significant (all *p* ≥ 0.505) (Table 2).

VAS analysis revealed a significant Group × Time interaction for appetite (F = 6.284, *p* = 0.017, ηp^2^ = 0.142). However, neither the main effect of Time (F = 0.335, *p* = 0.566) nor the main effect of Group (F = 0.556, *p* = 0.461) was significant. Appetite scores increased from 4.20 ± 1.58 to 4.70 ± 1.75 in the control group, whereas they decreased from 4.50 ± 1.28 to 3.70 ± 2.08 in the experimental group, indicating that the changes in appetite over time differed significantly between the groups (Table 3).

DEBQ analysis revealed no significant Group × Time interactions for restrained eating (F = 0.011, *p* = 0.917, ηp^2^ = 0.000), emotional eating (F = 1.273, *p* = 0.266, ηp^2^ = 0.032), or external eating (F = 2.541, *p* = 0.119, ηp^2^ = 0.063). For restrained eating, neither the main effect of Time (F = 0.011, *p* = 0.917) nor the main effect of Group (F = 0.261, *p* = 0.612) was significant. Emotional eating also showed no significant main effect of Time (F = 1.555, *p* = 0.220) or Group (F = 1.464, *p* = 0.234). Although emotional eating scores decreased from 24.05 ± 9.86 to 22.05 ± 7.86 in the experimental group, the change over time did not differ significantly between the groups. For external eating, the main effect of Time was not significant (F = 0.015, *p* = 0.903), whereas a significant main effect of Group was observed (F = 12.202, *p* = 0.001). However, the Group × Time interaction was not significant, indicating that changes in external eating over time did not differ significantly between the groups (Table 3).

**Table 2:** Descriptive statistics and repeated-measures ANOVA (Group × Time) for EEG and Stroop outcomes.

Outcome	Control Group		Experimental Group		Time	Group	Group × Time	ηp^2^ (G × T)
	Pre	Post	Pre	Post				
	Mean ± SD				F (*p*)	F (*p*)	F (*p*)	
Alpha (log)	−1.697 ± 0.224	−1.610 ± 0.164	−1.619 ± 0.205	−1.505 ± 0.130	8.767 (0.005)	3.753 (0.060)	0.159 (0.693)	0.004
Theta (log)	−1.291 ± 0.342	−1.426 ± 0.246	−1.305 ± 0.392	−1.571 ± 0.283	14.946 (<0.001)	0.819 (0.371)	1.592 (0.215)	0.040
Stroop RT low-cal (ms)	1423.15 ± 354.01	1276.00 ± 319.31	1439.20 ± 239.66	1234.05 ± 151.48	67.687 (<0.001)	0.001 (0.970)	3.139 (0.084)	0.076
Stroop RT high-cal (ms)	1402.45 ± 296.11	1289.45 ± 274.70	1451.75 ± 268.49	1253.95 ± 208.59				
Stroop ACC low-cal	29.90 ± 0.31	29.80 ± 0.62	29.40 ± 0.99	29.75 ± 0.64	2.496 (0.122)	5.621 (0.023)	2.496 (0.122)	0.062
Stroop ACC high-cal	29.85 ± 0.37	29.95 ± 0.22	29.50 ± 0.95	29.75 ± 0.55				

Values are presented as mean ± SD. EEG alpha/theta relative power values were natural-log transformed and do not directly represent raw EEG amplitudes. RT = reaction time (ms); ACC = accuracy. statistics shown collapsed across the calorie condition (Calorie and all Calorie interactions were non-significant, *p* ≥ 0.505). ηp^2^ = partial eta-squared for the Group × Time interaction. df = (1, 38) for all effects. Significance was set at *p* < 0.05; see footnote to Table 4 regarding correction for multiple comparisons.

**Table 3:** Descriptive statistics and repeated-measures ANOVA (Group × Time) for appetite (VAS) and eating behavior (DEBQ) outcomes.

Outcome	Control Group		Experimental Group		Time	Group	Group × Time	ηp^2^ (G × T)
	Pre	Post	Pre	Post				
	Mean ± SD				F (*p*)	F (*p*)	F (*p*)	
VAS appetite	4.20 ± 1.58	4.70 ± 1.75	4.50 ± 1.28	3.70 ± 2.08	0.335 (0.566)	0.556 (0.461)	6.284 (0.017)	0.142
DEBQ Restrained	23.25 ± 8.63	23.15 ± 8.82	24.70 ± 10.58	24.70 ± 9.46	0.011 (0.917)	0.261 (0.612)	0.011 (0.917)	0.000
DEBQ Emotional	26.50 ± 9.06	26.40 ± 10.16	24.05 ± 9.86	22.05 ± 7.86	1.555 (0.220)	1.464 (0.234)	1.273 (0.266)	0.032
DEBQ External	35.10 ± 7.53	34.05 ± 8.75	26.30 ± 6.57	27.20 ± 6.26	0.015 (0.903)	12.202 (0.001)	2.541 (0.119)	0.063

Values are mean ± SD. VAS = Visual Analogue Scale for appetite (0–10); DEBQ = Dutch Eating Behavior Questionnaire (Restrained, Emotional, External subscales). F and *p* are from 2 (Group) × 2 (Time) repeated-measures ANOVA; ηp^2^ = partial eta-squared for the Group × Time interaction; df = (1, 38). A significant Group × Time interaction was found only for VAS appetite (*p* = 0.017). The Group main effect for DEBQ-External reflects a pre-existing baseline difference between groups rather than an intervention effect. Significance was set at *p* < 0.05; see footnote to Table 4 regarding correction for multiple comparisons.

To control for multiple comparisons across the eight Group × Time interaction tests, a Bonferroni-adjusted significance threshold of α = 0.00625 was applied. At the uncorrected significance level, only VAS appetite showed a significant Group × Time interaction (F = 6.284, *p* = 0.017, ηp^2^ = 0.142), whereas the other seven outcomes showed no significant interactions. However, the VAS appetite interaction did not meet the Bonferroni-adjusted significance threshold, and none of the eight Group × Time interactions remained statistically significant after correction (Table 4).

**Table 4:** Summary of Group × Time interaction effects for all outcomes, with correction for multiple comparisons.

Outcome	F (1, 38)	*p* (Uncorrected)	ηp^2^	Significant After Bonferroni?	Interpretation
EEG Alpha (log)	0.159	0.693	0.004	No	No between-group difference
EEG Theta (log)	1.592	0.215	0.040	No	No between-group difference
Stroop RT	3.139	0.084	0.076	No	No between-group difference
Stroop ACC	2.496	0.122	0.062	No	No between-group difference
VAS appetite	6.284	0.017	0.142	No	Uncorrected *p* < 0.05; not significant after correction
DEBQ Restrained	0.011	0.917	0.000	No	No between-group difference
DEBQ Emotional	1.273	0.266	0.032	No	No between-group difference
DEBQ External	2.541	0.119	0.063	No	No between-group difference

Group × Time interaction from 2 (Group) × 2 (Time) repeated-measures ANOVA (for Stroop RT/ACC, the Group × Time term of the 2 × 2 × 2 model). ηp^2^ = partial eta squared. Bonferroni-corrected significance threshold across the eight interaction tests: α = 0.05/8 = 0.00625. No Group × Time interaction remained statistically significant after correction. The VAS appetite interaction was significant at the uncorrected level (*p* = 0.017) and is therefore reported as the strongest, but exploratory, between-group finding.

## 4. Discussion

The present study examined whether adding moderate-intensity cycling to a food-related Go/No-Go task produced greater acute changes in inhibitory control, frontal EEG activity, subjective appetite, and eating-related responses than the cognitive task alone in healthy young adults. The most prominent descriptive trend was observed for subjective appetite, which decreased in the experimental group but increased in the control group. However, this Group × Time interaction was significant only before Bonferroni correction, and no significant intervention-specific effects were detected for EEG, Stroop performance, or DEBQ outcomes. These findings suggest a possible acute subjective appetite response, but this interpretation remains preliminary and requires confirmation in adequately powered studies.

EEG analysis revealed significant main effects of Time for both alpha and theta power, with alpha power increasing and theta power decreasing from pre- to post-test in both groups. However, the Group × Time interactions were not significant, indicating that the magnitude of these changes did not differ between the experimental and control groups. Therefore, the observed EEG changes do not provide clear evidence of an effect specifically attributable to the addition of moderate-intensity cycling. Although previous studies have suggested that combined physical and cognitive tasks may influence prefrontal activity [21], evidence supporting their superiority over single-task conditions remains inconclusive [12]. Accordingly, the observed EEG patterns should be interpreted cautiously as exploratory findings rather than as confirmatory evidence of an intervention-specific neurophysiological effect.

Stroop reaction time decreased significantly from pre- to post-test in both groups, whereas the Group × Time interaction was not significant. Although acute exercise has been associated with improvements in cognitive performance in previous meta-analytic research [10], the comparable reduction observed in both groups indicates that the present change was not specific to the addition of cycling. Furthermore, although the food images and trial order were randomized at each assessment, the same Stroop task and stimulus set were administered at both time points. Randomization altered the presentation order but did not eliminate repeated exposure to the same stimuli and task structure. Therefore, the comparable reduction in reaction time observed in both groups likely reflects, at least in part, a practice effect rather than an intervention-specific improvement in inhibitory control or cognitive processing speed [28]. Because the potential practice effect associated with repeated use of the same stimulus set was not sufficiently anticipated when the study protocol was developed, a matched parallel version was not prepared. Stroop accuracy did not change significantly over time, and the Group × Time interaction was also not significant. In addition, neither the calorie condition nor any interaction involving calorie condition was significant. Overall, the findings do not provide clear evidence that adding moderate-intensity cycling improved Stroop performance beyond the food-related Go/No-Go task alone. Future studies should use parallel versions of the Stroop task with equivalent but non-overlapping food-image sets at the pre- and post-test assessments to reduce the influence of repeated testing.

The VAS appetite outcome showed a contrasting descriptive pattern between the groups, with appetite decreasing in the experimental group but increasing in the control group. This pattern is consistent with previous findings that acute exercise may transiently alter subjective appetite and energy-intake regulation [22]. However, although the Group × Time interaction was significant at the uncorrected level, it did not remain significant after Bonferroni correction and should therefore be interpreted cautiously. In addition, the VAS assesses subjective appetite sensations [17] rather than actual food intake or food choice. Accordingly, the present result should be regarded as a preliminary indication of a possible short-term appetite response rather than definitive evidence of an intervention-specific effect on subjective appetite. Future studies should standardize pre-assessment conditions and include objective measures of subsequent food intake or food choice.

None of the DEBQ subscales showed a significant Group × Time interaction, indicating that restrained, emotional, and external eating did not change differently between the experimental and control groups. Although emotional eating scores decreased descriptively in the experimental group, this change was not significantly greater than that observed in the control group and should therefore not be interpreted as an intervention-related improvement. The significant Group main effect for external eating likely reflects a pre-existing difference between the groups rather than an effect of the intervention, given the nonsignificant Group × Time interaction. Overall, the findings do not provide evidence that adding moderate-intensity cycling produced an acute improvement in self-reported eating behavior. The DEBQ was included as an exploratory secondary outcome to examine whether any immediate changes in self-reported eating-related tendencies could be detected. However, because the DEBQ primarily assesses relatively stable restrained, emotional, and external eating tendencies [16] rather than acute state changes, its use as a pre–post outcome measure for a single 20 min intervention represents a conceptual limitation and may partly explain the absence of significant interaction effects.

This study has several limitations. First, the sample size was relatively small, and the participants were limited to healthy young adults. Therefore, the sample did not adequately represent individuals with overweight or obesity, who are key target populations for weight-management and obesity-prevention interventions, and the findings should not be directly generalized to these populations. In addition, because this study involved a single acute intervention lasting approximately 20 min, the findings cannot be extended to sustained changes in subjective appetite, long-term changes in eating behavior, weight management, or obesity prevention. In addition, because the a priori sample-size calculation was based on an assumed large effect that was not empirically grounded, and because the sensitivity analysis indicated that only Group × Time interactions of f ≥ 0.23 (ηp^2^ ≈ 0.05) could be reliably detected, the study was underpowered for effects of the magnitude actually observed in most outcomes (Group × Time ηp^2^ = 0.000–0.142). The present findings should therefore be regarded as preliminary, and adequately powered studies are required to determine whether small intervention-specific effects exist. Second, because the Stroop task was administered before and after the intervention, repeated performance of the same task paradigm may have produced a practice effect, despite the randomization of the food images and trial order. Appetite and eating-related responses were also assessed using the self-reported VAS and DEBQ, whereas objective outcomes such as actual energy intake, food choice, and post-intervention food consumption were not measured. Furthermore, because the VAS and DEBQ were completed during preparation for EEG recording, stress or discomfort associated with electrode attachment, as well as environmental distractions, may have influenced participants’ subjective appetite ratings and DEBQ responses. This should be considered a potential limitation when interpreting these self-reported outcomes. Third, although participants were instructed to consume a meal 1–2 h before testing and to avoid arriving in a fasted state, the exact timing and content of the last meal were not standardized or recorded, and adherence to these instructions was not objectively verified. Caffeine intake, sleep quality, physical activity during the preceding 24 h, menstrual-cycle phase in female participants, and the time of data collection were also not controlled or recorded. These uncontrolled lifestyle and physiological factors may have influenced cognitive performance, EEG activity, and subjective appetite responses. Fourth, although participants rested for 5–10 min after exercise to allow perspiration to subside and were instructed to minimize unnecessary movements during EEG recording, residual effects of scalp perspiration, eye blinks, ocular activity, body or facial movements, and exercise-related physiological changes could not be completely excluded. Because approximately 40 s of relatively stable EEG data were manually selected and no automated artifact rejection or correction was applied, the possibility that residual artifacts or experimenter selection bias affected the alpha- and theta-power estimates cannot be ruled out. Accordingly, the observed EEG changes cannot be assumed to reflect exclusively neurophysiological changes and should be interpreted cautiously as exploratory findings. Finally, exercise intensity was regulated solely using self-reported RPE, with a target of approximately 12. Heart rate, cycle resistance, pedaling cadence, and actual workload were not objectively recorded. Therefore, although the same RPE-based adjustment procedure was applied to all participants, we could not objectively verify that each participant exercised within the moderate-intensity range or quantify the actual exercise dose. Individual differences in perceived exertion and actual exercise dose may have introduced variability in the intervention, limited its reproducibility, and affected the internal validity of the between-group comparison. Future studies should address these limitations by including larger and more diverse samples, implementing longer-term interventions, standardizing assessment conditions, objectively monitoring exercise intensity using heart rate, cycle resistance, pedaling cadence, and workload, and incorporating objective measures of food intake.

Overall, the present findings do not demonstrate intervention-specific improvements in frontal EEG activity, inhibitory control, or self-reported eating behavior. The divergent pattern observed in VAS appetite suggests a possible short-term subjective appetite response associated with the combined intervention. However, because the Group × Time interaction did not remain significant after Bonferroni correction and neither actual food intake nor food choice was assessed, this finding should be considered preliminary. Accordingly, the interpretation of this single-session study should be limited to a potential short-term subjective appetite response. Whether the intervention can produce sustained changes in subjective appetite or contribute to weight management or obesity prevention remains unconfirmed and should be examined in adequately powered, longer-term studies incorporating objective measures of food intake, food choice, and weight-related outcomes.

## 5. Conclusions

In this single-session study of healthy young adults, no statistically significant intervention-specific effects were observed for the primary outcomes of Stroop performance or frontal EEG alpha and theta power. Although subjective appetite showed contrasting changes between the groups, this pattern remained exploratory because the interaction did not remain significant after correction for multiple comparisons. Adequately powered longitudinal studies involving repeated interventions and individuals with overweight, obesity, or other clinical conditions are needed to confirm these findings.

## Figures and Tables

**Table 1:** General characteristics of the participants.

	Control Group (*n* = 20)	Experimental Group (*n* = 20)	*p*-Value
Sex (male/female)	12/8	9/11	0.342
Age (years)	20.75 ± 0.79	21.10 ± 1.41	0.339
Height (cm)	170.80 ± 7.53	168.40 ± 7.72	0.326
Weight (kg)	70.40 ± 15.70	66.65 ± 15.13	0.447

mean ± standard deviation.

## Data Availability

The data presented in this study are available on reasonable request from the corresponding author. The data are not publicly available due to privacy and ethical restrictions, as they contain potentially identifiable participant-level information, including behavioral and EEG-related data collected from human participants.

## Abbreviations

The following abbreviations are used in this manuscript:

ACC	Accuracy
BMI	Body mass index
DEBQ	Dutch Eating Behavior Questionnaire
EEG	Electroencephalography
RPE	Rating of perceived exertion
RT	Reaction time
SD	Standard deviation
VAS	Visual analogue scale
